# Early Surgical Intervention Improves Survival in Acute Intestinal Ischemia in the Intensive Care Unit

**DOI:** 10.1155/2021/6672591

**Published:** 2021-05-17

**Authors:** Hassan Adnan Bukhari, Anand Kumar

**Affiliations:** ^1^Department of Surgery, Umm AL-Qura University, Makkah, Saudi Arabia; ^2^University of Manitoba, Manitoba, Canada

## Abstract

The study is aimed at assessing whether the early surgical intervention improves survival in acute mesenteric ischemia with septic shock. A retrospective study design was applied to review the charts of patients admitted to the intensive care unit. The data were collected through a review of the full patient chart including physician and nursing notes, pathology reports, intraoperative findings, CT findings, and endoscopy. The diagnosis of AMI for each patient was determined through clinical presentation/endoscopic visualization/laboratory results/radiographic imaging, surgical exam (tissue or visual) and/or autopsy. Death and survival were evaluated between short and long-time-interval for septic shock groups using the chi-square test followed by calculating the *P* value. Total survival among the surgery group was 60 patients (95.24%) compared to 3 (4.76%) survival among patients who did not have surgery. The time from the onset of a shock to the time of surgical incision was calculated. The mean time to surgery was 17.7 hours. Total 65 patients (29.52%) had surgery between 4 and 12 hours from the onset of hypotension. Survivals among this group of patients were 41.7% (*n* = 25). The survival difference was statistically significant than died patients with respect to the time of surgical intervention (*P* = <0.001). Early removal of ischemic bowel in patients with AII-related surgery has improved survival.

## 1. Introduction

Acute mesenteric ischemia (AMI) defines as the prevalence of a sudden cessation of the mesenteric blood flow with the progression of symptoms that may differ from minutes to hours in time of onset [1]. It is usually an underdiagnosed reason for acute abdomen and is yet followed by a high mortality and morbidity rate [2]. Mortality from acute intestinal ischemia (AII) remains high despite the remarkable improvement in intensive care management and advanced imaging and intervention. For the last two decades, mortality remained very high at 60-80% [3, 4]. Almost 10% of the patients over 70 years of age were more prevalent to the AMI, since different comorbidities in elderly subjects may be related to an increased cardiovascular risk [2, 5, 6].

Recently, the survival rate has improved in patients with AMI. In a few series, survival rate ranged between 40% and 80% depending on the causes of AMI [7]. However, mortality may reach 100% especially when the systolic blood pressure drops below 70 mmHg [8, 9]. The gold standard diagnostic procedure in AMI is computed tomography with angiography (CTA) with an expected sensitivity and specificity of 91% and 99%, respectively [10].

Attempts have been made to improve survival in this group of patients. Translocation of bacteria is strongly related to septic shock in these patients in the presence of ischemic segment of bowel and release of endotoxins and free radicals [3]. Since the removal of sepsis sources helps in controlling the status of shock, surgical resection of the dead bowel is the mainstay therapy for AMI. Early surgical intervention including revascularization or bowel resection in patients with AII has improved survival [11].

Certain laboratory values may predict prognosis in patients with AMI [12–15]. A key role is also indicated in the literature on the inflammatory cascade, especially of inflammatory molecules such as interleukin 18, capable of causing the genesis of fibrosis and parenchymal abnormalities [16]. The correlation between these laboratory values and improving survival is unknown. The treatment and diagnosis of AMI depend on timely multidisciplinary management such as gastroenterologists, radiologists, surgeons, and intensive care physicians [17]. The diagnosis of AMI is usually complicated in critically ill patients, specifically for nonocclusive mesenteric ischemia (NOMI). It is monitored in the presence of clinical deterioration related to biological manifestations and digestive symptoms indicative of profound acute cell lysis or tissue ischemia [18]. One of the cornerstones of the diagnostic strategy is contrast-enhanced abdominal CT scan, which may offer direct or indirect arguments for impaired bowel vascularization [19]. On the contrary, the accuracy of the diagnosis of NOMI is questionable in critically ill patients. Direct visualization of the digestive tract is involved to confirm and evaluate the degree of necrosis [20].

The evaluation of the preoperative probability of mesenteric ischemia and the probabilities of surgical treatment is an essential area of improvement in the disorder management, considering the repeated diagnostic uncertainty of nonocclusive AMI in critically ill patients. In this regard, this study assesses whether the early surgical intervention improves survival in acute mesenteric ischemia with septic shock.

## 2. Materials and Methods

### 2.1. Study Design

The study has retrospectively reviewed the charts of 327 patients who were admitted to the intensive care unit (ICU) in 22 tertiary hospitals. The inclusion period was ranged from 1990 to 2007 to analyze the charts based on the onset of septic shock, time of surgical intervention, and outcome. Patients were included if they presented AMI during their ICU stay and were admitted to the ICU. AMI diagnosis was made using at least one of the six procedures: pathology report, intraoperative findings, a CT finding suggestive of ischemia that includes pneumatosis, mesenteric vessel occlusion, or gas, thickened nonenhanced loop of bowel, portal vein air, angiographic evidence of mesenteric occlusion, endoscopy, or autopsy finding. Patients were excluded if age ≤ 18 years at diagnosis, ≥50% of missing data in the patient's record, ischemia by extrinsic compression of mesenteric vessels, and invalidation of the diagnosis by autopsy or surgery. An autopsy was performed during admission time in ICU. Patients with acute mesenteric ischemia were not considered from the venous origin.

### 2.2. AMI Management

The AMI diagnostic modality depends on a multidisciplinary approach, which involves gastroenterologists, surgeons, radiologists, and physicians. The diagnosis of AMI was usually based on lower or upper digestive symptoms such as gastrointestinal hemorrhage, diarrhea, occlusion, feeding intolerance, organ failures, and biological appearances of tissue ischemia. Upper and lower endoscopic explorations or laparotomy and contrast-enhanced abdominal tomodensitometry were used for investigating a moderate to high likelihood for AMI. If localized bowel necrosis occurred, intestinal resection would be detected. If intestinal resection was detected, the researcher has investigated whether the diagnosis of AMI was identified by autopsy. The CT features were identified based on the protocol of radiologist. The study has also contributed if laparotomy contributed to the diagnosis and time from diagnosis to laparotomy. The researcher has also reported and discussed the major steps of managing patients with AMI including transferring patients to the operating room, decisions of withholding, and indications for major surgery.

### 2.3. Diagnostic Criteria

All abdominal CT scans were reviewed by two radiologists who were blinded from the final diagnosis. Considering intravenous contrast medium infusion, multidetector CT scans were initially conducted without contrast and secondarily delayed and early acquisitions. All patients were assessed for related comorbidities: peritonitis, sepsis, and single or multiorgan failure in terms of diagnosis process. The selection for rapid laparotomy was made for ascites infection, organ failure, and persistent abdominal tenderness. A 64-section multidetector CT scanner was used to perform the CT scan with an unimproved abdominal scan at the portal and arterial phases by 2 sequences for arteriography. Each patient was assessed through axial and reconstructed images for incomplete or complete vascular occlusion, abnormal mucosal enhancement, ascites, peritoneal enhancement, evidence of intestinal wall thickening, stranding ascites, mesenteric stranding, bowel dilation, portomesentric gas, and pneumoperitoneum for angiographic evidence.

Early AMI was defined in the absence of peritonitis, organ failure, and increased blood lactate levels on CT scan. Delayed AMI was defined in the presence of approximately one of the abovementioned criteria. All resected small-bowel specimens were used to obtain histologic confirmation of acute ischemic damage. A combination of angioplasty, intra-arterial vasodilation, thromboaspiration, stenting, and digestive arterial stenosis thrombolysis is based on the presence of the underlying and distal arteries, and the length of the vascular occlusion was included in radiologic revascularization.

Patients with organ failure, peritonitis or radiologic revascularization, and abdominal tenderness were recommended surgery by radiologists. In the presence of surgical revascularization of the superior mesenteric surgery, peritoneal lavage, anatomic assessment of small-bowel viability, and resection of necrotic small bowel, the radiologist has performed a midline laparotomy.

### 2.4. Data Collection

Regardless of the cause of AMI, the study focused on patients with AMI in whom the diagnosis was made based on (1) pathology report, (2) intraoperative finding, (3) CT finding suggestive of ischemia that includes pneumatosis, mesenteric vessels occlusion or gas, thickened nonenhanced loop of bowel, and portal vein air (60-70 s), (4) angiographic evidence of mesenteric occlusion, (5) endoscopy, or (6) autopsy finding indicating AMI. Patient demographics, vital signs including the onset of hypotension, laboratory values, survival to hospital discharge, and the time of surgery were recorded.

**Figure 1:**
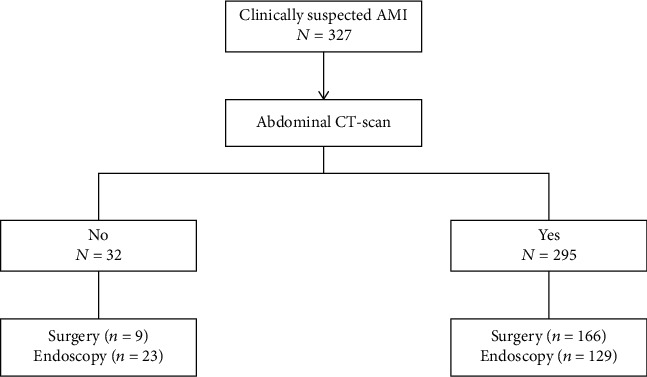
Study flowchart.

At the time of diagnosis, laboratory data were recorded such as for arterial lactate. The study has observed arterial lactate measurement 24 h after AMI diagnosis. The diagnostic procedures were reported for each patient. The study has investigated whether the diagnosis of AMI was performed through autopsy (Figure 1).

### 2.5. The Criteria for Septic Shock

Hypotension episode did not respond to two liters of crystalloid challenge or responded transiently for less than 1 hour to fluid challenge. Hypotension that responded to 2-liter fluid resuscitation with no subsequent deterioration in the patient's hemodynamics was not considered a septic shock-related hypotension. Ischemic bowel suggested by clinical suspicion and/or lactic acidosis was not considered sufficient for the diagnosis and was not included. Hypotension was defined as mean blood pressure below 65 mmHg, systolic blood pressure of <90 mmHg, or when the systolic blood pressure dropped by 40 mmHg from the patient's baseline consistent with SCCM/ACCP criteria for septic shock. Sepsis was diagnosed based on the presence of two or more of the following features: (1) heart rate of 90 bpm, (2) temperature high than 101°F or lower than 96.8°F, and (3) white blood cells higher than 10,000 or lower than 4500.

The time interval between the onset of septic shock-related hypotension and the time of source control (time of surgical incision) was evaluated. The source control time interval was divided into 0-4 hours, 4-12 hours, and more than 12 hours. Survival to hospital discharge was the main outcome.

### 2.6. Statistical Analysis

SPSS 21 software (SPSS software [IBM Corp. 2011 IBM SPSS Statistics for Windows, Version 21.0 Armonk, NY, USA: IBM Corp]) was used to perform statistical analyses. Categorical variables were reported as percentages and analyzed using Fisher's exact test or chi-squared test. According to the distribution, continuous variables were expressed as mean with standard deviation (SD) or median with interquartile range (IQR).

Age, laboratory values 24 hours before and after source control, and survival to hospital discharge were analyzed. Univariate analysis was used to determine whether there is a difference between short versus long time interval groups. Death and survival were evaluated between short and long-time-interval for source control groups using the chi-square test followed by calculating the *P* value. A *P* value of less than 0.05 was considered statistically significant.

### 2.7. Ethical Considerations

The King Abdul-Aziz University Ethics Committee has provided institutional approval, and this study complied with the Helsinki Declaration. Patients' data were anonymously recorded in the final database for assuring confidentiality. A waiver was obtained for written informed consent due to the retrospective design of the study.

## 3. Results and Discussion

A total of 50.8% of females out of 327 patients were admitted to ICU. A total of 166 patients were identified with source control, out of which 27.8% underwent surgical intervention while 22.9% underwent medical treatment (Table 1), while remaining 43.9% of the patients were not identified with any source control/location. The 30-day survival period was far better than 90-day and 15-day survival periods (Table 1).

The mean length of stay (LOS) for the hospital was 19.65 ± 31.90 days while it was 8.00 ± 10.22 days for ICU (Table 1). The normal body temperature among patients admitted in ICU was ≤36 degrees (82.0%), which shows the presence of hypothermia. Only 7.3% of patients reported heart rate > 90 bpm, and 79.7% of patients reported respiratory rate > 20 bpm (Table 2). Heme was the major comorbidity reported among patients (2.11 ± 1.43), followed by hepatic and CNS (Table 3).

The study has identified 295 (90.21%) patients with AMI-related septic shock while the remaining 32 (9.79%) out of 327 patients were excluded because septic shock developed after the surgery. Survival to hospital discharge was 21.4% (70 patients). 295 patients had documented hypotension due to ischemic bowel (100%). Out of 295 patients, 166 patients (56.27%) underwent surgery and 129 (43.73%) did not undergo any surgical intervention. Total survival among the source control group was 60 patients (95.24%) compared to 3 (4.76%) survival among patients who did not have surgery (Table 4). The time from the onset of a shock to the time of surgical incision was calculated. The mean time to surgery was 17.7 hours with standard deviation (SD) of 27.37. Delaying surgery by surgical resection of the affected bowel resulted in poor prognosis. 43 patients (25.90%) underwent surgical resection within the first 4 hours from the onset of hypotension (Table 4).

In order to demonstrate a survival benefit among patients who underwent emergent surgical intervention, a 3 × 3 table was constructed. The time interval was divided into <4 hours, 4-12 hours, and more than 12 hours. 43 (25.90%) patients underwent surgical therapy in less than 4 hours with a survival rate of approximately 35.0% (*n* = 21). 65 patients (29.52%) had surgery between 4 and 12 hours from the onset of hypotension. Survivals among this group of patients were 41.7% (*n* = 25). Survival rate declined to 23.3% (*n* = 14) among patients in whom source control occurred after 12 hours (34.94%, *n* = 58). The difference in survival was statistically significant (*P* = <0.001) as shown in Table 5.

This retrospective study has demonstrated that early surgical resection of ischemic bowel is a critical determinant of survival to hospital discharge in AMI-related septic shock. Diagnosis of ischemic bowel remains a huge challenge to surgeons and physicians especially in the presence of septic shock. No screening tool has high sensitivity, and even the CT angiogram has a sensitivity of 64% and specificity of 92% [21]. This will result in delaying the surgical intervention. The presence of shock and stabilizing the patient is another major determinant in delaying the surgical intervention because time will be spent to make the patient stable enough to undergo a major surgical procedure.

A high mortality rate and intestinal failure in survivors are associated with AMI, which is considered as an abdominal emergency [22]. A systematic review of 3692 AMI patients has indicated 100% untreated patients and 70% inhospital death rates [15]. Another study has reported an inhospital survival rate that underwent endovascular revascularization. According to the findings, the inhospital survival rate was reported among 36% in patients as compared with 50% in cases of conventional treatment [23]. If revascularization was performed in early AMI, it is likely to get the best survival rates as compared to this study [24].

Lack of diagnostic imaging makes the diagnosis of ischemic bowel even more challenging. Few studies concluded that the shorter the time interval between the onset of symptoms and surgical management, the higher the chance to survive [4, 7–11]. Calculating the time interval using the onset of symptoms is very difficult because usually, the symptoms could be nonspecific and insidious. Abdominal pain is the most common presenting symptom which is not specific and could be very mild initially [9]. Evidence lacks to address determinants of survival in the presence of septic shock in combination with AMI. Therefore, it is difficult to assess the significance of the present results and observations with previous studies.

It is important to mention the weaknesses of this study, since it is a retrospective study with limitations. Different types of bias may occur during data collection. The onset of hypotension is not always accurate because the first documented hypotension is used to calculate the time interval which might not be the true onset. However, the prospective study could not be conducted due to the scarcity of this disease entity and randomization is impossible. Secondly, the occurrence of comorbidities may have been underestimated as they have been reported from the charts of patients. The study has estimated that most of the comorbidities were consistently and reliably consigned in the medical files, making the bias minimal regardless of chronic inflammatory disease and hypercoagulability.

AMI remains a therapeutic and diagnostic challenge in critically ill patients. The diagnostic contribution of the abdominal CT scan is restricted in this study. Undisputed indications for surgical intervention are represented from radiological signs of advanced-stage ischemia for evaluating the extent of bowel necrosis and the probability of intestinal resection. The lack of radiological signs indicated that mesenteric ischemia should not be considered for ruling out the diagnosis and still confirms additional digestive explorations by laparotomy and endoscopy in case of high clinical suspicion. Early surgery by removal of ischemic bowel in patients with AMI-related septic shock has improved survival. As the time from the onset of hypotension to surgery increases, the survival rate will decline incrementally.

## Figures and Tables

**Table 1:** Patient characteristics.

Characteristics	AMI	
Male (*n*, %)	161	49.2%
Female (*n*, %)	166	50.8%
Location (*n* = 166)		
Surgery	91	27.8%
Medical	75	22.9%
Height (cm)	167.53 ± 12.91 cm	
Weight (kg)	76.10 ± 19.77	
BMI (kg/m^2^)	27.07 ± 6.10	
Lactate mg/dL	6.62 ± 5.06	
Survival duration (days)	29.40 ± 40.32	
Survival at 15 days	215	65.7%
Survival at 30 days	253	77.4%
Survival at 90 days	239	73.1%
Length of stay (days)		
ICU LOS	8.00 ± 10.22	
HOSP LOS	19.65 ± 31.90	
Hospital days preshock	8.04 ± 18.626	
Score		
Total apache	28.18 ± 9.41	

ICU: intensive care unit; HOSP: hospital; LOS: length of stay.

**Table 2:** Clinical characteristics.

Characteristics	
Temperature (degrees)	
≤36 degrees	242 (82.0%)
>36 degrees	53 (18.0%)
Heart rate (bpm)	
>90 bpm	24 (7.3%)
Respiratory rate (bpm)	
>20 bpm	222 (79.7%)
CO_2_ (ppm) (mean ± SD)	35.4235 ± 10.32491
WBC (mean ± SD)	16.1523 ± 12.23678

CO_2_: carbon dioxide; PCO_2_: partial pressure of carbon dioxide; WBC: white blood cells.

**Table 3:** Organ failure.

	Mean	Std. deviation
Renal insufficiency (mean ± SD) (range)	1.1435	.61376
Respiratory disease (mean ± SD) (range)	1.1242	.54104
Heme (mean ± SD) (range)	2.1183	1.43849
Metabolic (mean ± SD) (range)	1.2160	.58884
CNS (mean ± SD) (range)	1.4388	1.22838
Hepatic (mean ± SD) (range)	1.9684	1.53299
COAG (mean ± SD) (range)	1.4109	.96428

CNS: central nervous system; COAG: coagulation tests.

**Table 4:** Patient's survival with AMI-related septic shock.

Characteristics	AMI (*n* = 295)
Survival to discharge	
Yes	70 (21.40%)
No	225 (68.80%)
Hypotension	
Yes	295
No	0
Source control	
Surgical intervention	166 (56.27%)
No surgical intervention	129 (43.73%)
Source control survival (*n* = 63)	
Yes	60 (95.24%)
No	3 (4.76%)
Source control (time)	17.7 ± 27.37 hours
Surgical resection time (*n* = 166)	
<4 hours	43 (25.90%)
4-12 hours	65 (39.16%)
>12 hours	58 (34.94%)

**Table 5:** Univariate analysis for survival difference.

Time of surgical intervention	Survived	Died	Total	*P* value
<4 hours	21 (35.0%)	22 (20.75%)	43 (25.90%)	<0.001
4-12 hours	25 (41.7%)	40 (37.74%)	65 (29.52%)	
>12 hours	14 (23.3%)	44 (41.50%)	58 (34.94%)	
Total	60	106	166	

## Data Availability

The datasets used and analyzed during the current study are available from the corresponding author on reasonable request.
